# Kidney Failure and Abdominal Discomfort as Initial Signs of Extramedullary Acute Myelogenous Leukemia

**DOI:** 10.3390/clinpract11030061

**Published:** 2021-07-22

**Authors:** Peter Ferkis Steinfeld, Thomas Knoop, Linn Hereide Trovik, Hilde Kollsete Gjelberg, Torjan Magne Haslerud, Håkon Reikvam

**Affiliations:** 1 Department of Medicine, Haukeland University Hospital, N-5021 Bergen, Norway; Peter.Ferkis.Steinfeld@helse-bergen.no (P.F.S.); Thomas.Knoop@helse-bergen.no (T.K.); linn.hereide.trovik@helse-bergen.no (L.H.T.); 2 Institute of Medical Science, University of Bergen, N-5021 Bergen, Norway; 3 Department of Pathology, Haukeland University Hospital, N-5021 Bergen, Norway; Hilde.Kollsete.Gjelberg@helse-bergen.no; 4 Department of Radiology and Nuclear Medicine, Haukeland University Hospital, N-5021 Bergen, Norway; Torjan.Magne.Haslerud@helse-bergen.no; 5 Institute of Clinical Science, University of Bergen, N-5021 Bergen, Norway

**Keywords:** kidney failure, hydronephrosis, acute myelogenous leukemia, PET

## Abstract

Although rare, acute myelogenous leukemia (AML) can include extramedullary manifestations, sometimes presenting as a solid tumor called a myeloid sarcoma. Myeloid sarcoma can be the cause of the initial presenting complaint before AML diagnosis, or may be detected as a sign of disease-relapse after treatment. Here, we report a case in which the initial presentation included abdominal discomfort and signs of kidney failure. Further investigation revealed signs of unilateral hydronephrosis. Due to a diagnostic delay, the patient was diagnosed with AML with extramedullary manifestation only after the development of full-blown leukemia. Biopsy of the compressive tumor confirmed an extramedullary myeloid sarcoma, and [^18^F]-FDG-PET/CT proved useful for patient diagnosis and follow-up. This case report illustrates the importance of thorough examination and diagnosis, as a serious underlying disease with a rare cause can debut with an unusual presentation.

## 1. Introduction

Traditionally defined as a decreased glomerular filtration rate, renal failure clinically manifests as abrupt and sustained increases in the serum levels of urea and creatinine, potentially disrupting salt and water homeostasis [1]. Renal failure is a frequent occurrence in patients with malignant disease. Although such renal failure is often considered multifactorial, it is clinically useful to classify the cause as prerenal, intrinsic, or postrenal. Prerenal failure is common and is often caused by dehydration or general circulatory involvement. Intrinsic renal failure often occurs due to renal parenchymal invasion by solid or hematologic cancers, and usually does not involve prominent clinical sequelae. Lymphomas and leukemias are the cancers most commonly linked to intrinsic renal failure, with a high incidence discovered at autopsy among patients with disseminated lymphoma [2]. Multiple myeloma (MM)-related renal failure is a specific entity that includes cast nephropathy, amyloidosis, and light-chain nephropathy [3]. MM patients are also at risk for hypercalcemia-associated renal failure. Postrenal causes of renal failure are more common among cancer patients than in the general population, and obstruction may occur at any level of the urogenital tract. Solid tumors of the prostate, bladder, uterus, and uterine cervix are common causes of obstruction, and can lead to hydronephrosis. On the other hand, hematological malignancies rarely lead to hydronephrosis and renal failure—especially myeloid malignancies, which seldom involve extramedullary sites [4].

In the present paper, we discuss the initial manifestations of a patient presenting with renal failure and abdominal discomfort. The diagnostic work-up revealed an uncommon presentation of acute myelogenous leukemia (AML).

## 2. Case Presentation

A 30-year-old woman was referred to the nephrology department due to kidney failure. Her previous medical history included dietary advice for irritable bowel syndrome (IBS) and abnormal cervical smears, verified by cone biopsy to be cervical intraepithelial neoplasia grade III (CIN 3), and were followed-up by a gynecologist every six months. She had suffered two silent miscarriages, most recently just three months before the consultation. Testing performed in the setting of the recent miscarriage revealed an elevated serum creatinine level of 129 µmol/L (normal range 45–90 µmol/L). Her lactate dehydrogenase (LDH) level was also slightly elevated to 228 U/L (normal range 105–205 U/L). Testing for occult fecal blood was positive, while a urine test strip yielded negative results. At the same time, the patient began to experience severe pain in the lower left quadrant of abdomen and left flank. Based on these signs and symptoms, her family doctor referred her to the nephrology department. Before her appointment in the outpatient clinic, ultrasound of her kidneys and urinary tract revealed normal-sized kidneys, dilation of the left renal pelvis, and a mass on the right ovary. Reduced visibility to the posterior abdominal wall by examination was noted.

At the primary consultation, the patient was in a good general condition. Her blood pressure was 140/90 mmHg, and she had no abdominal pain or swelling in her extremities. Auscultation of her heart and lungs was normal. The urine strip test was negative, urine microscopy revealed physiological urine, and no proteinuria was detected.

**Figure 1:**
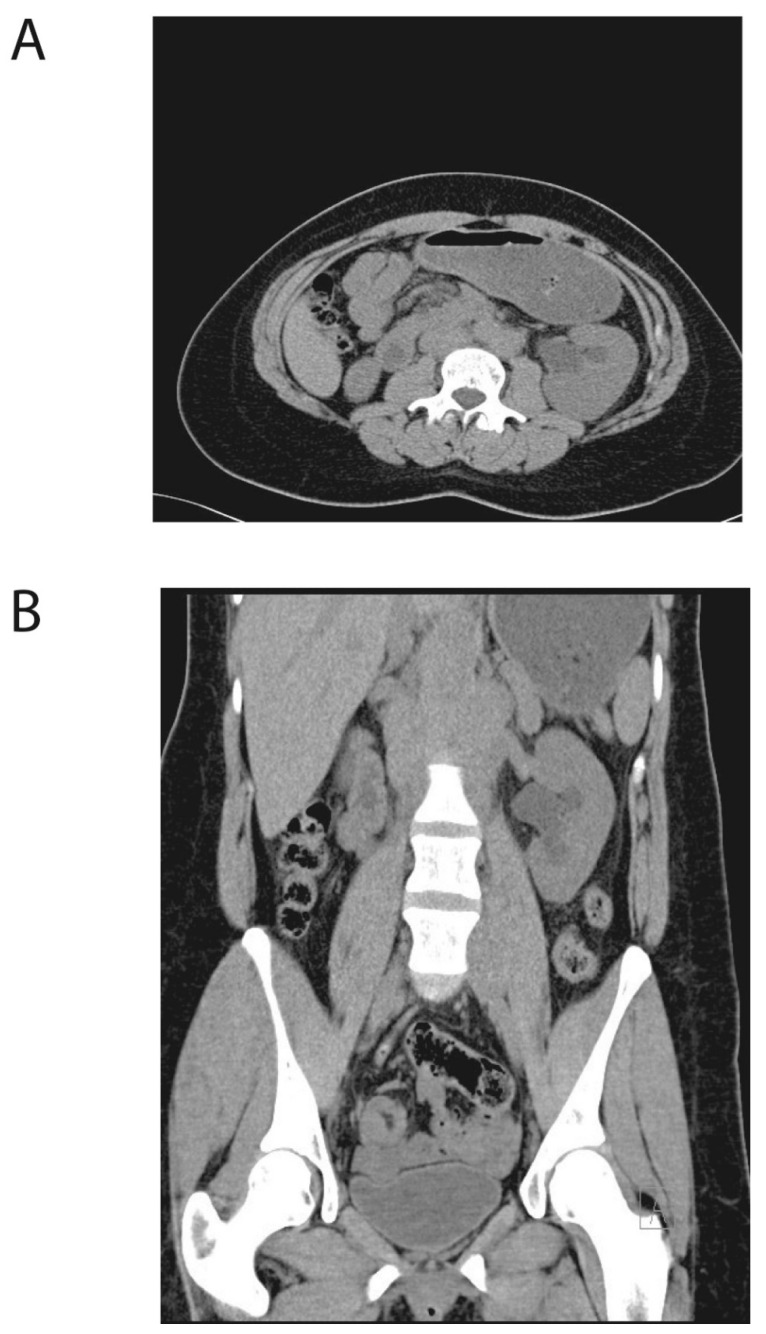
Initial computed tomography (CT) scan of the urinary tract. (**A**) Transversal plan and (**B**) frontal plan. The initial CT of the patient’s abdomen, revealing normal kidney parenchyma. The renal pelvis of the left kidney shows dilation compared to right side. The left ureter is dilated and S-shaped near the renal pelvis.

Blood tests revealed slight kidney failure, with a creatinine level of 121 µmol/L, and low-grade anemia, with a hemoglobin (Hb) level of 11.5 g/dL (normal range, 11.7–15.3 g/dL). Plasma renin concentration was 82.5 μIU/mL (normal range, 4.4–46.1 μIU/mL) and serum aldosterone was 931 pmol/L (normal range, 20–620 pmol/L). Based on these findings of kidney failure, along with left-sided hydronephrosis and high plasma renin and aldosterone concentrations, the patient was referred for computerized tomography (CT) scanning of the urinary tract. CT scanning revealed no masses or stones, and both kidneys had a normal parenchyma. However, the renal pelvis of the left kidney was still dilated compared to the right side. The left ureter was also dilated and S-shaped near the renal pelvis, but without the typical appearance of a ureteropelvic junction obstruction (Figure 1). Potential pathology on the posterior abdominal wall or in other parenchymal organ was noticed to have reduced sensitivity on this examination. The patient also underwent colonoscopy, with normal findings.

**Figure 2:**
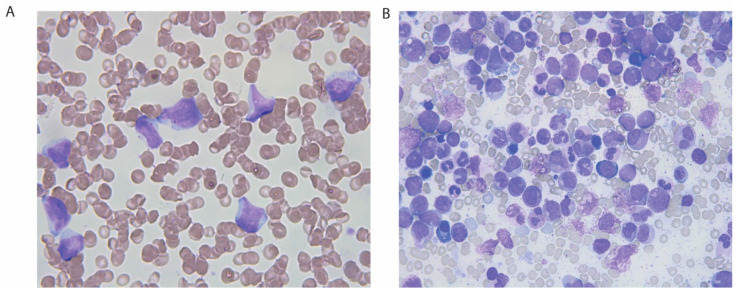
Blood and bone marrow smear, hematoxylin and eosin-stained sections. (**A**) Blood smear showing leukocytosis with a left shift, myelomonocytic blasts, and some more immature blasts. (**B**) Marrow aspirate demonstrating hypercellular marrow with dysplastic maturation of the myeloid and erythroid cell lines, myelomonocytic blasts, and myeloblasts.

After one month, the patient returned to the outpatient clinic for follow-up. Her creatinine was 118 µmol/L, and her anemia had worsened (Hb, 9.7 g/dL). Strikingly, the patient also showed thrombocytopenia with a platelet count of 42 × 10^9^/L (normal range, 165–387 × 10^9^/L), and leukocytosis with a white blood cell count (WBC) of 16.4 × 10^9^/L (normal range, 3.5–11.0 × 10^9^/L). A blood smear revealed leukocytosis with a left shift, granulocyte dysplasia, and ~30% immature blasts (Figure 2). On the same day, the patient was admitted to the hematological department with suspected acute leukemia. At admission, she had lower back pain, although was otherwise asymptomatic and had normal organ status. Microscopy of bone marrow aspirate revealed hypercellular marrow with all cell lines in place, dysplastic maturation of the myeloid and erythroid cell lines, and 36% myeloblasts (Figure 2). The findings were considered to be compatible with AML with subclassification; acute myelomonocytic leukemia, French American British (FAB) classification M4. This was also confirmed by immunophenotyping by multiparametric flow cytometry.

These findings were verified by immunophenotyping via multiparametric flow cytometry. Real-time polymerase chain reaction (RT-PCR) analysis revealed *CBFB-MYH11* fusion. Chromosomal G-banding analysis demonstrated an inversion of chromosome 16; inv(16) (p13.1q22).

**Figure 3:**
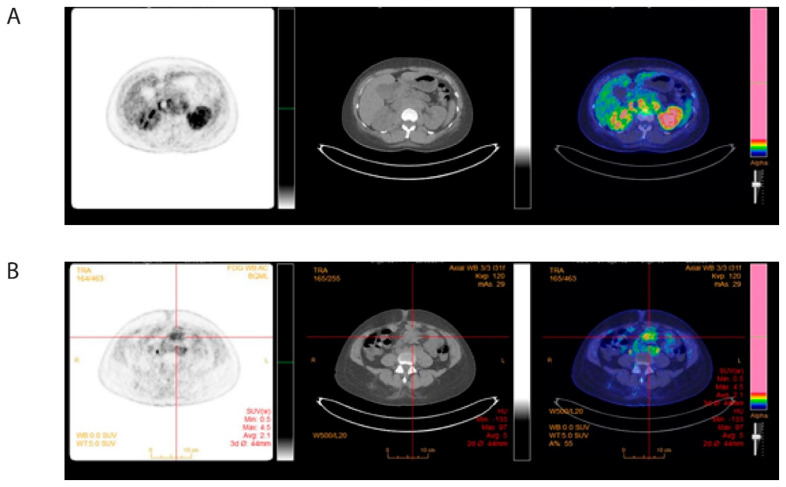
Pretreatment 18-fluorodeoxyglucose (FDG) positron emission tomography (PET)-computed tomography (CT) of abdomen. (**A**) [^18^F]-FDG-PET/CT at diagnosis showing FDG-positive para-aortic (**B**) and mesenteric tumor masses.

A new CT scan indicated retroperitoneal masses along the major arteries. Further diagnostic work-up included [^18^F]-fluorodeoxyglucose (FDG) positron emission tomography (PET)/CT, which demonstrated increased metabolism (SUVmax: 6.1) in para-aortal and mesenteric masses, strengthening the suspicion of abdominal tumor masses (Figure 3).

**Figure 4:**
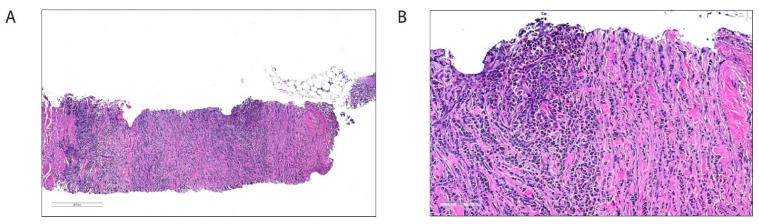
Histologic images of the biopsy from the retroperitoneal tumor masses, hematoxylin and eosin-stained sections. (**A**) Soft tissue with diffuse infiltration of tumor cells between striated muscle and connective tissue fibers (H&E, approx. 180 times magnification). (**B**) A higher magnification reveals tumor cells somewhat affected by crush artifacts, with immature and mature eosinophils (H&E, approx. 580 times magnification).

**Figure 5:**
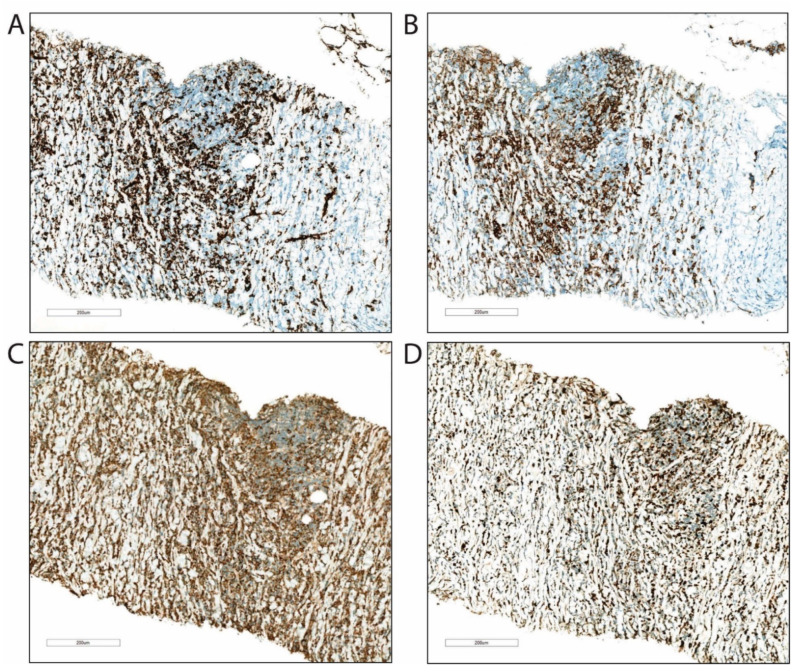
Immunohistochemical examination of the biopsy from the retroperitoneal tumor masses. (**A**) The majority of the tumor cells stained positive for myeloid stem cell markers CD34 (**B**) and CD117, (**C**) and for markers associated with cells of myeloid origin, myeloperoxidase (**D**) and lysozyme. Proliferation marker Ki-67 (MIB1) demonstrates high cell proliferation.

These results suggested that the increased creatinine levels and hydronephrosis were due to compression of the left ureter caused by the tumor masses. To verify tumor infiltration, an ultrasound-guided biopsy from the retroperitoneum was performed. Histopathological analysis confirmed infiltration of the soft tissue by immature myeloid cells, consistent with myeloid sarcoma (Figure 4). The majority of the tumor cells expressed myeloid markers myeloperoxidase (MPO) and lysozyme, together with hematopoietic stem cell markers CD117 and CD34. A distinct subpopulation of tumor cells expressed monocytoid markers CD68 and CD163 together with myeloperoxidase (MPO), and lacked CD34 positivity, suggesting myelomonocytic type of AML (Figure 5).

**Figure 6:**
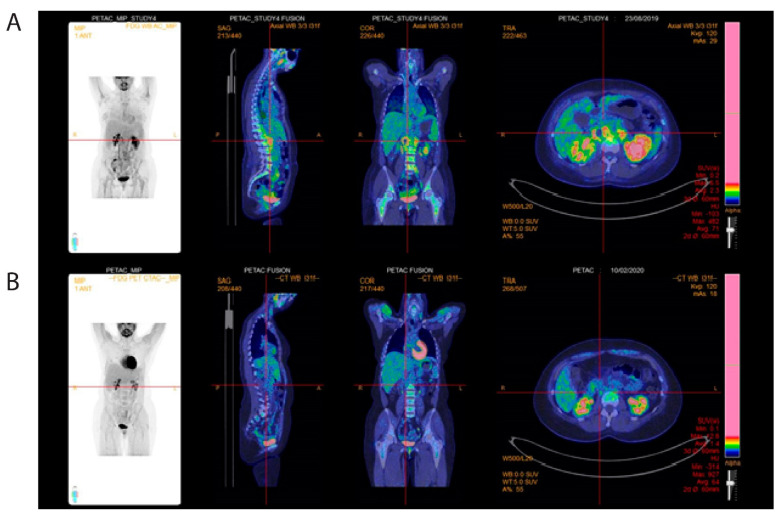
Posttreatment 18-fluorodeoxyglucose (FDG) positron emission tomography (PET)-computed tomography (CT) of abdomen. (**A**) [^18^F]-FDG-PET/CT pre-therapeutic FDG-positive para-aortic masses. (**B**) After treatment showing metabolic complete remission.

A 10 F pyelostomy catheter was placed to decompress the kidney, and then treatment was initiated using the standard AML protocol of idarubicin and cytarabine (the 3 + 7 regimen) [5]. Within one week after chemotherapy initiation, creatinine levels were normalized, and the pyelostomy catheter was removed. No signs of tumor lysis were observed. After the first induction cycle, the patient exhibited complete remission, defined as normalization of hematological parameters without transfusions, and bone marrow blasts <5% [5]. Consolidation treatment was administered with three consecutive cycles of high-dose cytarabine [6]. A new FDG-PET/CT after the end of treatment confirmed the treatment success, showing metabolic regression of the masses in the retroperitoneum (Figure 6), and regression of the myeloid sarcoma at the abdominal wall. At 12 months after diagnosis, the patient is currently in complete remission, without signs of kidney failure.

## 3. Discussion

In the present case, the primary presentation of AML included abdominal discomfort and kidney failure. Hydronephrosis is caused by obstruction anywhere in the urogenital tract, which results in renal pelvis dilation, halted drainage, and subsequent renal failure [7]. Such a finding should always prompt a diagnostic work-up, with investigation for causes for urine obstruction. Tumors of the abdominal wall, a known cause of ureter obstruction and hydronephrosis, are uncommon and can be difficult to visualize with different imaging modalities. In cases with concomitant diagnosis of renal failure and hydronephrosis, and without findings of other intrinsic causes of renal failure, further diagnostic work-up is required, including radiological imaging, to identify the cause of hydronephrosis and to potentially alleviate the obstruction of urine flow. In our present case, diagnosis was delayed because no further precautions had been taken, and a complete investigation for renal failure and hydonephrosis was not carried out. Once further diagnostic work-up was initiated by a nephrologist, the patient already exhibited full-blown leukemia with significant blood and bone marrow infiltration.

AML is a highly malignant disease, resulting in proliferation and a lack of differentiation of hematological stem and precursor cells [8]. The disease is usually localized to the bone marrow, where malignant clone overgrowth leads to nominal hematopoiesis interference and suppression, resulting in bone marrow failure with anemia, thrombocytopenia, and leukopenia [8]. However, this disease can also involve extra-medullary manifestations, and exhibit a growth pattern that resembles solid tumors [4]. Extramedullary manifestations of AML, known as myeloid sarcomas or chloromas [9], can present in a wide range of organ systems, including the gastrointestinal [10], musculoskeletal [11], and cardiovascular systems [12]. Due to the heterogeneity of manifestations and the potential to affect such a wide variety of organ systems, it is important to consider it as a possible differential diagnosis—especially if other underlying causes are not immediately obvious. Although myeloid sarcomas can develop in any organ or site in the body, they most commonly occur at immunological sanctuary sites, e.g., the testis, ovary, and central nervous system [4,9,13]. Certain AML subgroups more frequently debut with extramedullary disease, and apparent predictive features include AML with maturation (FAB M4 and M5) and cytogenetic anomalies, such as inv(16)/t(16;16) or t(8;21) [4,13,14]. Our present patient exhibited both acute myelomonocytic leukemia (FAB M4) and cytogenetic aberration with inv(16). Monocytoid differentiation was demonstrated both by, immunophenotyping of bone marrow, CD14 and CD64, and immunohistochemistry of tumor, CD68 and CD163. Notably, extramedullary presentation of AML is also relatively frequent in relapsing disease, particularly after allogeneic stem cell transplantation [15,16].

There is a lack of clinical trials investigating myeloid sarcoma treatment [13]. However, treatment with standard AML chemotherapy is usually administered based on biological rationale, experience, and general recommendations. Among cases in which isolated myeloid sarcomas were treated only for the local complication, a high proportion later progressed to AML [9].

The prognostic impact of extramedullary disease is controversial [9]. However, most recent and larger studies support an equal or even better prognosis for patients with extramedullary disease [17,18]. Organ manifestation may impact outcome, although probably cytogenetic and molecular genetic findings are of greater importance [19].

During the last two decades, FDG-PET/CT has emerged as a powerful and highly specialized diagnostic imaging tool. Metabolic imaging utilizing FDG-PET/CT substantially contributes to the diagnosis and treatment of different types of malignant disease, and for hematological malignancies especially for lymphoma [20]. In contrast, the exact role of FDG-PET/CT in leukemia, and particularly in extramedullary disease, has not been fully established [9]. However, the present case clearly illustrates the benefits and opportunities inherent with this modality. In our present case, FDG-PET/CT was successfully used for the initial diagnostic work-up for the manifestation of extramedullary disease, as well as for evaluating the treatment response and follow-up.

Overall, our present case illustrates the importance of prompt and thorough investigation of kidney failure and hydronephrosis in a young and previously healthy individual. In rare cases, the underlying cause may be compromising malignant tumor growth, and the detected disease may be potentially curable.
